# Functional analysis of bovine interleukin-10 receptor alpha in response to *Mycobacterium avium* subsp. *paratuberculosis* lysate using CRISPR/Cas9

**DOI:** 10.1186/s12863-020-00925-4

**Published:** 2020-11-02

**Authors:** Sanjay Mallikarjunappa, Umesh K. Shandilya, Ankita Sharma, Kristen Lamers, Nathalie Bissonnette, Niel A. Karrow, Kieran G. Meade

**Affiliations:** 1 Animal and Bioscience Research Department, Animal and Grassland Research and Innovation Centre, Teagasc, Grange, Co. Meath, Ireland; 2 Centre for Genetic Improvement of Livestock, Department of Animal Biosciences, University of Guelph, Guelph, Ontario N1G 2W1 Canada; 3 Agriculture and Agri-Food Canada, Sherbrooke Research and Development Centre, Sherbrooke, QC J1M 0C8 Canada

**Keywords:** Johne’s disease (JD), Interleukin-10 receptor alpha gene (*IL10RA*), CRISPR/cas9, Gene knockout, Candidate gene

## Abstract

**Background:**

The interleukin-10 receptor alpha (*IL10RA)* gene codes for the alpha chain of the IL-10 receptor which binds the cytokine IL-10. IL-10 is an anti-inflammatory cytokine with immunoregulatory function during the pathogenesis of many inflammatory disorders in livestock, including Johne’s disease (JD). JD is a chronic enteritis in cattle caused by *Mycobacterium avium* subsp. *paratuberculosis* (MAP) and is responsible for significant economic losses to the dairy industry. Several candidate genes including *IL10RA* have been found to be associated with JD. The aim of this study was to better understand the functional significance of *IL10RA* in the context of immune stimulation with MAP cell wall lysate.

**Results:**

An *IL10RA* knock out (KO) bovine mammary epithelial cell (MAC-T) line was generated using the CRISPR/cas9 (Clustered Regularly Interspaced Short Palindromic Repeats/CRISPR-associated protein 9) gene editing system. These *IL10RA* KO cells were stimulated with the immune stimulant MAP lysate +/− IL-10, or with LPS as a positive control. In comparison to unedited cells, relative quantification of immune-related genes after stimulation revealed that knocking out *IL10RA* resulted in upregulation of pro-inflammatory cytokine gene expression (*TNFA*, *IL1A*, *IL1B* and *IL6*) and downregulation of suppressor of cytokine signaling 3 (*SOCS3)*, a negative regulator of pro-inflammatory cytokine signaling. At the protein level knocking out *IL10RA* also resulted in upregulation of inflammatory cytokines - TNF*-α* and IL-6 and chemokines - IL-8, CCL2 and CCL4, relative to unedited cells.

**Conclusions:**

The findings of this study illustrate the broad and significant effects of knocking out the *IL10RA* gene in enhancing pro-inflammatory cytokine expression and further support the immunoregulatory role of *IL10RA* in eliciting an anti-inflammatory response as well as its potential functional involvement during the immune response associated with JD.

## Background

Infection with the bacteria *Mycobacterium avium subsp. paratuberculosis* (MAP) causes Johne’s disease (JD), a chronic enteritis in cattle. JD is highly contagious with significant economic and animal welfare implications to the dairy industry [1, 2]. MAP is considered a pathogen with zoonotic potential, although this remains controversial [3]. JD is prevalent worldwide and is difficult to control due to lack of availability of treatment options and the absence of an efficacious vaccine to prevent MAP infection [4].

Over the years, much emphasis has been placed on understanding the genetic basis of susceptibility to JD in cattle [5]; and this has revealed the heritable nature of MAP infection and led to the identification of single nucleotide polymorphisms (SNPs) across the bovine genome associated with JD phenotypes [6]. Associations between several candidate genes and JD have since been reported [7–14]. While these candidate gene studies reported statistical associations with various phenotypes, they have not confirmed their functional relevance with regards to the host response to MAP infection and/or the pathology of JD.

Previously, Verschoor et al. reported a strong association of SNPs in the interleukin-10 receptor alpha gene (*IL10RA*) with MAP infection status in dairy cattle [14], and also mastitis [15], another economically important inflammatory disease of cattle. This gene codes for the alpha chain of the IL-10 receptor whose ligand, IL-10, functions as a key regulator of inflammation and has been implicated in the pathogenesis of MAP infection. Polymorphisms in human *IL10RA* have also been associated with IBD, and human immune cells lacking a functional IL-10 receptor have been found to be non-responsive to anti-inflammatory negative feedback signals provided by IL-10, and as a result, contributed to gut inflammation [16].

The objective of this study was to determine the biological and functional significance of bovine *IL10RA* by creating *IL10RA* knockout (KO) epithelial cell line. In the first step, the CRISPR/Cas9 gene editing technique [17] was used to create an *IL10RA* KO bovine mammary epithelial cell line (MAC-T). MAC-T cells were chosen because previous studies have reported their ability to process MAP and to determine the course of MAP infection by enhancing phagocytic uptake of MAP by macrophages [18, 19]. MAC-T cells also express a variety of pattern-recognition receptors, such as TLR2 and TLR4, which make them responsive to pathogen-associated molecular patterns (PAMPs) such as bacterial lipopolysaccharide (LPS) [20]. Following confirmation of *IL10RA* KO, the MAC-T cells were stimulated with the immune stimulants MAP lysate +/− IL-10, or LPS as a positive control, and the expression of key immune genes was determined.

## Results

### Genome cleavage detection (GCD) assay and western blot analysis

**Fig. 1:**
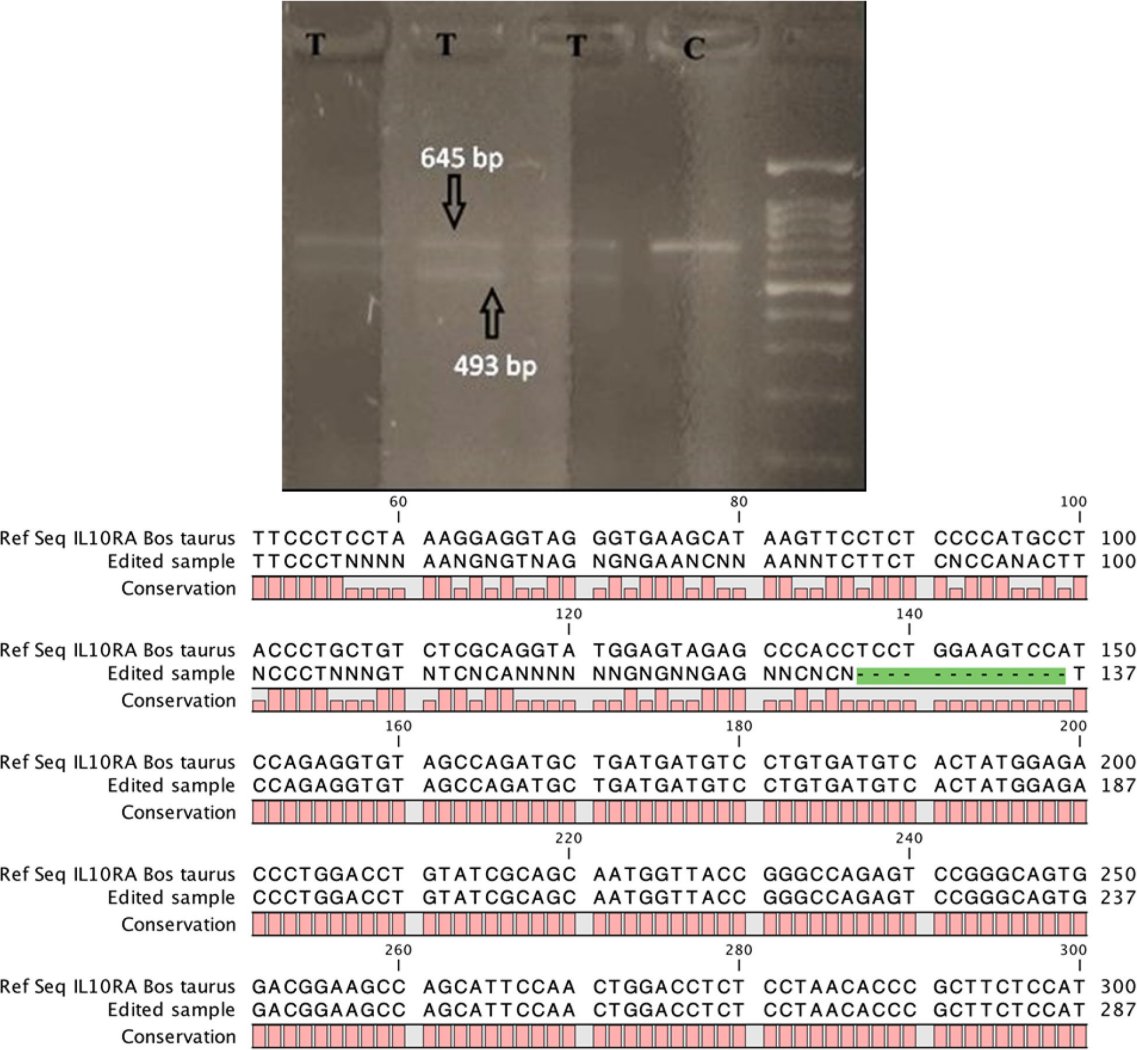
**a** Gel image of Genomic Cleavage Detection Assay using Bovine MAC-T transfected cells. Detection of two bands (upper parental 645 bp band and lower 493 bp cleavage band - shown with arrows) in transfected cells (T) as opposed to one parental band in control non-transfected cells (C) indicates gene editing in *IL10RA* by specific sgRNAs. **b** Aligned sequences of exon 3 of IL-10RA gene of knockout (KO) MAC-T cells with reference sequence showing 13 bp deletion using CLC Genomics Workbench

Induction of indels were detected in transfected cells (T) as determined by presence of two bands - upper parental band (645 bp) and lower cleavage band (493 bp) as opposed to one parental band (645 bp) in control non-transfected cells (C) (Fig. 1a). This indicates on-target genome editing due to non-homologous end joining (NHEJ) repair in sgRNA transfected MAC-T cells and in turn *IL10RA* KO in sgRNA transfected MAC-T cells. Sanger sequencing further revealed 13 bp deletion in exon 2 of *IL10RA* (Fig. 1b).

**Fig. 2:**
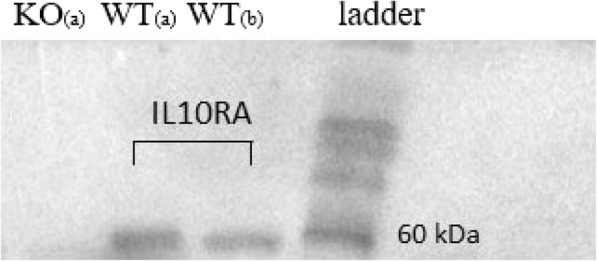
Western blot analysis indicating absence of IL10RA protein expression in *IL10RA* knockout (KO) MAC-T cells, while the expression was observed in unedited (WT) MAC-T cells (band at 60 kDa). **a** 40 μg of loaded total lysate protein; **b** 20 μg of loaded total lysate protein

The follow-up Western blot analysis revealed that IL10RA protein was undetected in KO cell lysate, while it was detected in WT MAC-T cell lysate (60 kDa protein); thereby validating the generation of *IL10RA* KO in MAC-T cells (Fig. 2).

### Real-time PCR quantification of target genes

**Fig. 3:**
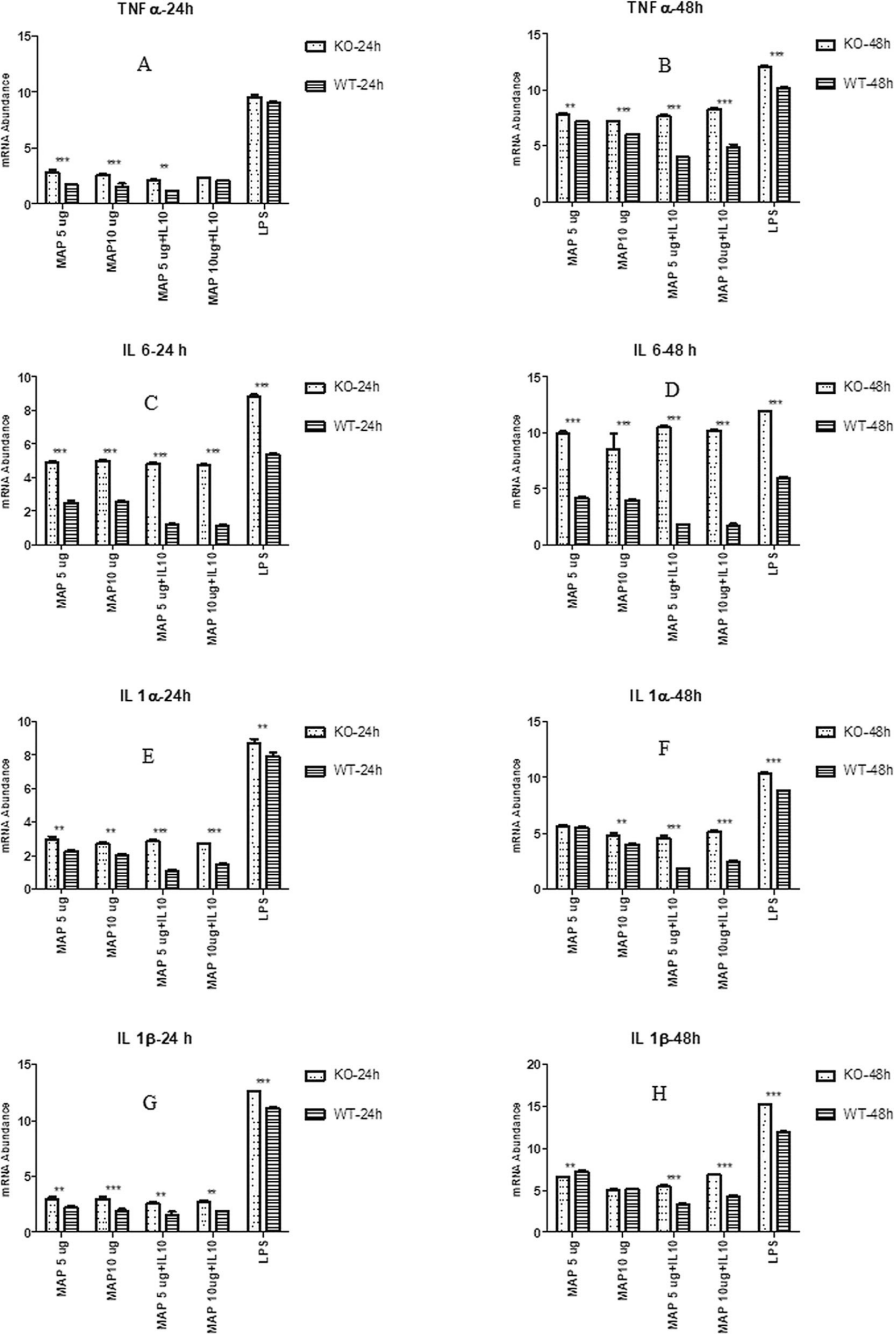
**a-h:** Relative mRNA transcript expression (delta CT values) of different pro-inflammatory cytokine genes *TNF-α* (A/B), *IL-6* (C/D), *IL-1α* (E/F) and *IL-1β* (G/H) in *IL10RA* KO MAC-T cells (KO) and unedited (WT) MAC-T cells following 24 and 48 h stimulation with MAP lysate, MAP lysate + IL10, or LPS (5 μg/ml). Values (mean ± SEM, *n* = 4 representing independent replicate experiments) with asterisks indicate a significant difference: *** denotes significant differences at *P* < 0.001;** denotes significant differences at *P* < 0.01

**Fig. 4:**
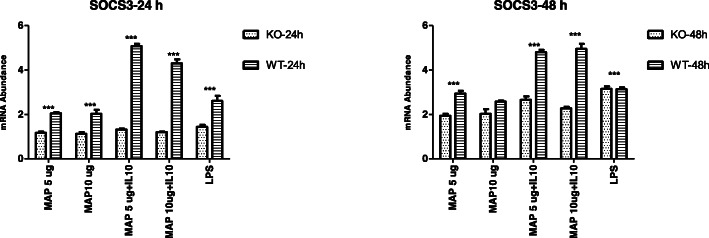
Relative mRNA transcript expression (delta CT values) of anti-inflammatory *SOCS3* in *IL10RA* KO MAC-T cells (KO) and unedited WT MAC-T cells following 24 h and 48 h stimulation with MAP lysate, MAP lysate + IL10, and LPS (5 μg/ml). Values (mean ± SEM, *n* = 4 representing independent replicate experiments) with asterisks indicate a significant difference: *** denotes significant differences at *P* < 0.001

The expression of pro-inflammatory cytokine genes *TNFA*, *IL1A*, *IL1B* and *IL6* was significantly higher (*P* < 0.05) in *IL10RA* KO cells compared to WT MAC T cells at 24 and 48 h post stimulation with MAP+/− IL-10, or LPS (Fig. 3 A-H) except in two instances; these included no difference in *TNFA* expression between the two cell types at the 24 h time point for LPS or combined MAP lysate (10 μg/ml) + IL-10 stimulation (50 μg/ml), and at the 48-h time point where no significant differences in *IL1B* and *SOCS3* expression were observed between the two cell types when stimulated with MAP lysate (10 μg/ml) or LPS. On the other hand, the expression of anti-inflammatory *SOCS*3 was significantly lower in the *IL10RA* KO than the WT MAC-T cells for the same immune stimulation with either MAP+/− IL-10, or LPS (Fig. 4).

**Fig. 5:**
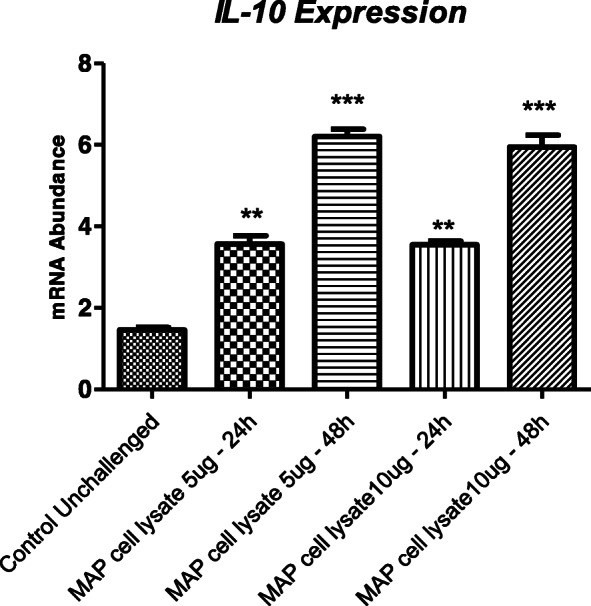
Relative *IL10* mRNA transcript expression (delta CT values) in WT MAC-T cells stimulated with MAP lysate (5 and 10 μg/ml) at two time points (24 and 48 h) and its comparison with unchallenged WT MAC-T cells (control unchallenged). Values (mean ± SEM, *n* = 4 representing independent replicate experiments) with asterisks indicate a significant difference: ** *p* < 0.01; *** *p* < 0.001

Expression of *IL-10* was significantly higher in WT MAC-T cells stimulated with MAP lysate (5 and 10 μg/ml) when compared with WT unstimulated MAC-T cells at 24 and 48 h time points (Fig. 5); this means *IL10* expression occurs in MAC-T cells in response to MAP lysate. The observed expression changes of components of the IL-10/IL10RA signaling pathway (*TNFA*, *IL1A*, *IL1B*, *IL6 and SOCS3*) in KO cells are therefore due to *IL10RA* KO even in the presence of increased *IL-10* expression.

### Cytokine/chemokine levels in culture supernatant

**Fig. 6:**
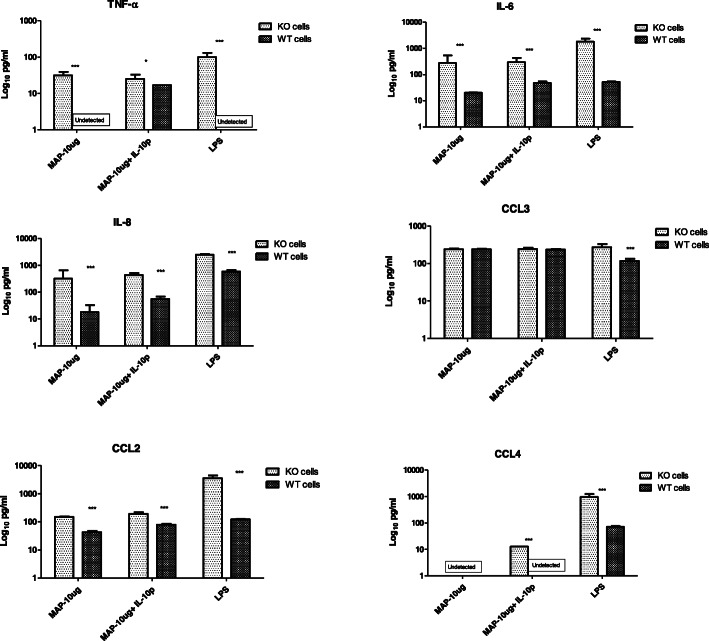
Concentration of cytokines measured by Multiplex Immunoassay in culture supernatant of *IL10RA* knockout MAC-T cells (KO cells) Vs unedited WT MACT cells. Values (mean ± SEM, *n* = 4 representing independent replicate experiments with asterisks indicate a significant difference: *** denotes significant differences at *P* < 0.001; ** denotes significant differences at *P* < 0.01; * denotes significant differences at *P* < 0.05

Expression of TNF-α, IL-6, IL-8, CCL2 and CCL4 proteins was also significantly elevated in *IL10RA* KO cells when compared with WT MAC-T cells at the 48-h time point post stimulation with MAP lysate +/− IL-10 or LPS. No difference in chemokine CCL3 expression was observed between the two cell types (Fig. 6).

## Discussion

IL-10 is a major anti-inflammatory cytokine secreted by macrophages, regulatory T cells, dendritic cells and some epithelial cells [21]. Upon binding of IL-10 to IL10RA homodimer, transmembrane signalling via IL10RB homodimer leads to the phosphorylation of the transcription factor Signal Transducer and Activator of Transcription 3 (STAT3); STAT3 induces expression of SOCS3 that subsequently inhibits the expression of cytokine genes *TNFA*, *IL1A*, *IL1B* and *IL6* [22]. Human studies have demonstrated that mutated IL-10 receptors on various immune cell types are unable to respond to IL-10 anti-inflammatory signaling and this was accompanied by increased amounts of TNF-α and other pro-inflammatory cytokines in response to co-stimulation with IL-10 and LPS, and attenuated amounts of SOCS3 in response to IL-10 [16].

The *IL-10, IL10RA* and *IL10RB* genes have been extensively studied as candidate genes in the context of human IBD. For example, SNPs in these genes have been reported in patients with early onset IBD [23]. More specifically, a SNP in *IL10RA* was found to be significantly associated with CD patients who tested positive for MAP infection [24]; detection of MAP in patients with CD has also been reported elsewhere [3, 25], but a cause-effect relationship remains to be established.

The *IL-10, IL10RA* and *IL10RB* genes have also been investigated as risk genes for JD. A previous study conducted by our group identified SNPs in *IL-10*, *IL10RA*, *IL10RB* and provided evidence that SNPs in *IL10RA* were associated with MAP-specific antibodies in dairy cattle [14]. As a follow-up to Verschoor et al. [14], the present study was designed to elucidate the functional role of *IL10RA* in the context of MAP immune challenge; this was achieved in vitro by creating a *IL10RA* KO MAC-T cell line and comparing the function of this *IL10RA* KO to unedited WT MAC-T cells stimulated with MAP lysate +/− IL-10, or LPS as a positive control. We utilized a CRISPR/cas9 based protein approach to create the *IL10RA* KO MAC-T cell line, which involved delivering ribonucleotide protein (RNP) complex consisting of the IL10RA gene specific sgRNA and cas9 protein. As expected, increased *IL-10* expression was observed following stimulation of WT MAC-T cells; this demonstrated that MAC-T cells are capable of expressing *IL-10* and that this gene is responsive to stimulation with MAP lysate. When *IL10RA* was knocked out, stimulation with MAP lysate (5 and 10 μg/ml) revealed significantly greater expression of the pro-inflammatory genes *TNFA*, *IL1A*, *IL1B* and *IL6.* Elevated expression was validated at the protein level for cytokines TNF-α and IL-6 and chemokines IL-8, CCL2 and CCL4 in comparison to the WT MAC-T cells. Similarly, increased pro-inflammatory cytokine gene and protein expression was also observed when *IL10RA* KO cells were stimulated with combined MAP lysate + IL-10. In concordance with these findings, the expression of anti-inflammatory *SOCS3*, which is an important regulator of pro-inflammatory cytokines, was lower in the KO cells compared to the WT cells. This increased pro-inflammatory cytokine/chemokine expression and attenuated anti-inflammatory *SOCS3* expression reflects the important role of the IL10RA receptor in regulating IL-10 signaling pathway; wherein, when *IL10RA* is knocked out, the ability of cells to respond to IL-10 stimulation and subsequent inhibition of pro-inflammatory cytokine/chemokine expression is lost.

Involvement of the IL-10-IL10R signaling pathway with regards to the host response to MAP infection and/or the pathology of JD is complex. Upregulation of the anti-inflammatory IL-10 response in MAP-infected animals has been shown to coincide with a shift from subclinical to clinical stage of infection characterized by higher antibody response in MAP infected animals [4], and Verschoor et al. reported that cattle with *IL10RA* haplotype (AGC) had a higher likelihood of being positive for MAP-specific antibodies [14]. The early protective immune response against MAP infection involves increased expression of the pro-inflammatory cytokines IFN-γ, TNF-α, and IL-12 that enhance anti-microbial activity of immune cells against MAP [26, 27]. Albeit protective in nature, prolonged pro-inflammatory cytokine secretion can also lead to chronic inflammation at the site of infection thereby contributing to immunopathology associated with JD [4, 28, 29]. In addition to this, MAP has evolved several strategies to promote its survival, and upregulation of immunoregulatory IL-10 is one such tactic [30–32]. Decreased IFN-γ and upregulated IL-10 for example, was detected in MAP-stimulated peripheral blood mononuclear cells (PBMCs) harvested from sub-clinically MAP-infected animals [33], and addition of exogenous IL-10 was shown to enhance MAP viability in infected PMBCs and monocyte-derived macrophages [34, 35]. Although our study did not investigate haplotype- or genotype-specific *IL10RA* effects or MAP-host cell interactions, the whole gene knockout approach gave us valuable insight into the anti-inflammatory role played by *IL10RA* and how responsiveness to MAP lysate is affected when *IL10RA* is knocked out.

Lastly, the current study used LPS as a positive control PAMP because its recognition by TLR4 [20], and subsequent activation of pro-inflammatory cytokines [36] and induction of the IL-10 signalling pathway has been well characterized [22, 37]. Since the response of *IL10RA* KO MAC-T cells to LPS largely paralleled the response to MAP lysate stimulation, this implies that *IL10RA* is important for regulating the host response to a variety of different PAMPs.

## Conclusions

Many studies are found in the literature reporting statistical associations between JD candidate genes and MAP infection status, further warranting the functional validation of these genes. Our study is the first to conduct a functional validation of *IL10RA*, one of the candidate genes for JD. We used CRISPR/CAS9 gene editing technique to create *IL10RA* KO MAC-T cell line. This whole gene knockout approach gave us valuable insight into the anti-inflammatory role played by *IL10RA* and how responsiveness to MAP lysate is affected when *IL10RA* is knocked out. The *IL10RA* KO cell line created in this study can also serve as a model to evaluate the anti-inflammatory response against different PAMPS.

## Methods

### Bovine mammary epithelial cell line (MAC-T cells)

MAC-T cells, developed by Huynh et al. [38], were used to perform *IL10RA* gene KO in vitro. MAC-T cells were cultured in T25 tissue culture flasks (Corning, Tewksbury, MA, USA) at 37 °C with 5% CO2 in Dulbecco’s Modified Eagle Medium (DMEM; Invitrogen, Burlington, ON, Canada) supplemented with 4.0 mM L-glutamine, 10% heat inactivated fetal bovine serum (FBS; Invitrogen), 25 mM HEPES buffer (Invitrogen), 0.25 μg/ml amphotericin B (Invitrogen), 1% Penicillin/Streptomycin (100 unit/ml of Penicillin and 100 μg/ml Streptomycin; Invitrogen), 1 mM Sodium Pyruvate (Invitrogen), and 5 μg/ml insulin-transferrin-selenium (Invitrogen).

### MAP cell lysate preparation

MAP cell lysate was provided by Dr. Brandon Plattner’s lab (University of Guelph, Canada). *Mycobacterium avium subsp. paratuberculosis* (MAP) GC86 strain (obtained from Dr. Lucy Mutharia (University of Guelph, Canada) and cultured in Middlebrook 7H9 broth (Sigma-Aldrich) to an optical density of 0.2 to 0.4 at 540 nm, then pelleted by centrifugation at 3500 x g for 20 min and washed twice with ice cold PBS. The MAP pellet was then suspended in PBS and sonicated on ice using a probe sonicator (Model 120, Fisher Scientific); with 60% amplitude for three cycles of 10 min bursts followed by a 10 min chilling period between cycles. MAP sonicate was then centrifuged at 12000 x g for 5 min, following which, the pellet was discarded and the lysate supernatant saved. The protein concentration of MAP lysate was determined using Bio-Rad protein assay (Richmond, CA, USA).

### Single guide RNA design (sgRNA)

Single guide RNA (sgRNAs) targeting bovine *IL10RA* were designed using the Synthego knockout guide RNA design tool (www.synthego.com). Three sgRNAs which were predicted to have with minimal off-target effects were selected (sgRNA1: auggaguagagcccaccucc; sgRNA2: ccucuggauggacuuccagg; and sgRNA3: caucuggcuacaccucugga) and custom synthesized by Synthego (Menlo Park, CA, USA). Out of three, only sgRNA2 that targets *IL10RA* exon 3 (www.ensembl.org - ENSBTAT00000006870) was used for transfection. Lyophilised sgRNAs were diluted in Tris-EDTA buffer to a final working concentration of 33.3 pmol/μL.

### Transfection of MAC-T cells

MAC-T cells were transfected with sgRNA and the cas9 protein complex using Lipofectamine CRISPRMAX (Invitrogen, Carlsbad, CA) reagent as per manufacturer’s instructions. Briefly, 24 h prior to transfection, 70,000 cells were seeded in a 24-well plate so that the wells were 70% confluent at the time of transfection. On the day of transfection, the sgRNA and cas9 protein complex (1.2:1 ratio) was added to the Lipofectamine CRISPMAX reagent. The sgRNA/cas9 lipid complex was incubated at room temperature for 15 min, after which it was added to each well to carry out transfection. The cells were then incubated for 48 h at 37 °C with 5% CO_2_.

### Genome cleavage detection assay

Post transfection, cells were harvested for subsequent sub-culture and detection of insertion or deletion of bases (indels) induced by the sgRNA-cas9 complex at the expected *IL10RA* site using GeneArt® Genomic Cleavage Detection Kit (Life Technologies, Carlsbad, CA) as per the manufacturer’s instructions. Briefly, the sgRNA transfected and non-transfected samples were PCR amplified using the same of set of primers (forward: GAATACCCTGAGGGCTGTATTG; reverse: GGCCCGATGCTGAGTATTTAT) flanking the region of interest. After re-annealing, samples were treated with and without detection enzyme (provided with GCD assay kit) and run on a 2% agarose gel to check induction of indels.

### Single cell clonal expansion

Mixed MAC-T cell populations in which indels were detected by genome cleavage assay were serially diluted in a 96-well plates to generate clonal populations. These monoclonal progeny cells were sub-cultured and further subjected to Sanger sequencing to identify indels in the *IL10RA* gene.

### Western blot analysis

Western blot was carried out to determine IL10RA protein expression in edited and unedited (WT) MAC-T cells. Edited and WT MAC-T cells were cultured in T25 flasks to approximately 70–80% confluence, pelleted by centrifugation at 400 X g for 15 min and lysed by sonication in ice cold radioimmunoprecipitation assay (RIPA) lysis buffer containing protease inhibitor (Thermofisher Scientific, Pierce, Rockford, IL). Following sonication, the lysate was incubated for 15 min followed by centrifugation at 13000 x g for 5 min at 4 °C to collect cell lysate supernatant for the Western blot analysis.

Cell lysate proteins were separated by subjecting them to 12% SDS-PAGE electrophoresis followed by their transfer to a 0.45 μm pore size polyvinylidene difluoride (PVDF) membrane (Millipore Inc., Burlington, CA, United States) using the iBlot 2 Dry Blotting System (Thermofisher Scientific). The PVDF membrane was later blocked with 5% (w/v) skim milk powder in 1X TBST buffer (0.3% Tris, 0.8% NaCl, 0.02% KCl, 0.1% Tween 20) for 1 h and then incubated with polyclonal rabbit anti-bovine IL10RA antibody (Antibodies-online Inc., Limerick, PA, USA) overnight at 4 °C. After washing the membrane three times for 5 min each in TBST, the membrane was incubated with anti-rabbit IgG secondary antibody conjugated to horseradish peroxidase (Antibodies-online Inc., Limerick, PA, USA) for 1 h at room temperature. Protein bands were visualized using an ECL Plus Western blotting system according to manufacturer’s instructions (Amersham Biosciences, Piscataway, NJ, United States).

### Gene expression study in *IL10RA* knockout MAC-T cells

Following confirmation of *IL10RA* knockout at protein level in edited CRISPR/cas9 MAC-T cells, hereon referred as *IL10RA* KO MAC-T cells, the biological relevance of *IL10RA* was studied by stimulating KO and WT MAC-T cells with various immune stimulants such as MAP lysate, MAP lysate plus recombinant bovine IL-10 (Cedarlane Labs, Burlington, ON), and LPS as a positive control (*Escherichia coli* serotype O111:B4, Sigma-Aldrich). The response to immune stimulation was compared between *IL10RA* KO and WT MAC-T cells by relative quantification of expression of various immune-related genes (Table 1) within the IL-10/IL10RA receptor signaling pathway using real-time polymerase chain reaction (RT-PCR).

**Table 1:** Summary of the designed primer sequences for gene expression analysis (F forward primer, R reverse primer)

Gene	Sequence	Amplicon size (bp)
*IL1A* - F	TTGGTGCACATGGCAAGTG	475
*IL1A* - R	GCACAGTCAAGGCTATTTTTCCA	
*IL1B* - F	GCCTTCAATAACTGTGGAACCAAT	100
*IL1B* -R	GTATATTTCAGGCTTGGTGAAAGGA	
*IL6* - F	GGCTCCCATGATTGTGGTAGTT	523
*IL6* -R	GCCCAGTGGACAGGTTTCTG	
*TNFA* - F	CGGTGGTGGGACTCGTATG	352
*TNFA* - R	CTGGTTGTCTTCCAGCTTCACA	
*SOCS3* - F	GCCACTCTCCAACATCTCTGT	97
*SOCS3* - R	TCCAGGAACTCCCGAATGG	
*IL10* - F	AAAGCCATGAGTGAGTTTGACA	155
*IL10* - R	TGGATTGGATTTCAGAGGTCTT	
*GAPDH* - F	TGGAAAGGCCATCACCATCT	60
*GAPDH* - R	CCCACTTGATGTTGGCAG	

In order to carry out this gene expression study, *IL10RA* KO MAC-T cells and WT MAC-T cells were seeded at 70,000 cells/well in two 24-well cell culture plates each and incubated overnight at 37 °C with 5% CO_2_. Both cell types were stimulated in quadruplicate for 24 and 48 h with MAP lysate (5 μg/ml and 10 μg/ml), MAP lysate + IL-10 (5 μg/ml and 10 μg/ml of MAP lysate; 50 μg/ml IL-10 protein), or LPS (5 μg/ml). The immune stimulant concentrations and analysis time points for MAP lysate and LPS were based on previous studies [20, 39, 40], and the concentration of IL-10 was determined based on a preliminary study conducted in our lab.

At 24 and 48 h post challenge, the corresponding time point plate wells were washed with PBS and cells were lysed using 250 μL Trizol (Invitrogen) per well. Total RNA was then extracted from Trizol cell lysate using RNeasy Mini Kit (Qiagen) as per the manufacturer’s instructions. The total RNA concentration was measured using Cytation 5 equipment (BioTek). From each sample, total RNA (500 ng) was reverse transcribed to cDNA using a High-Capacity cDNA Reverse Transcription Kit (Applied Biosystems).

### Primer design and real-time PCR

The primers used in the gene expression study were designed using the Primer3Plus software (www.primer3plus.com [41];). A list of primers used, and their sequences, are provided in Table 1. Relative quantitative expression of target genes was determined using the StepOnePlus™ Real-Time PCR System (Applied Biosystems, Burlington, ON, Canada). Real-time PCR was performed using SYBR® Green qPCR supermix and the PCR conditions consisted of 50 °C for 2 min, 95 °C for 2 min, 40 cycles of 95 °C for 15 s, 58.3 °C for 30 s and 72 °C for 30 s. Dissociation curves were generated at the end of amplification to ensure the presence of single amplified products. The cycle threshold (**Ct**) values for each sample were obtained by StepOne Plus software. Glyceraldehyde 3-phosphate dehydrogenase (*GAPDH*) and β2-Microglobulin (*B2M*) were the two genes initially tested as house-keeping genes. Because of its stable expression in both *IL10RA* KO and WT MAC-T cells for all immune stimulants, only *GAPDH* was used further to normalise the expression of target genes using first derivative (delta ct) method [42, 43].

All experiments were carried out in quadruplicate to compare the differences in mRNA expression levels. The delta CT values of genes Tumor Necrosis Factor alpha (*TNFA*), Interleukin 1 alpha (*IL1A*), Interleukin 1 beta (*IL1B*), Interleukin 6 (*IL6*) and Suppressor of cytokine signaling 3 (*SOCS3*) were analyzed using two-way ANOVA test followed by the Bonferroni test, and a *p*-value of ≤0.05 was considered statistically significant using Graphpad Prism version 4.00 (GraphPad Software, 2003, San Diego California, USA). All data were presented as the mean ± the standard error of the mean (SEM).

Relative *IL10* expression in WT MAC-T cells stimulated with MAP lysate (5 and 10 μg/ml) was compared with unchallenged WT MAC-T cells only at 24 and 48 h time points. *IL-10* expression levels were analysed by Dunnett’s test and a *p*-value of ≤0.05 was considered statistically significant. Data were presented as the mean ± SEM.

### Cytokine/chemokine analysis of culture supernatant from IL10RA knockout and WT MAC-T cells

Following immune stimulation as described above, culture supernatant from each replicate well was harvested at 48 h and stored at − 80 °C. Measurement of cytokines (TNF-α, IL-6, IL-8) and CCL chemokines (CCL2, CCL3, CCL4) in cell culture supernatants from both cell types was performed using a customised Bovine Cytokine/Chemokine Magnetic Bead Panel (Millipore Corporation, Billerica, MA, USA) as per manufacturer’s instructions. The assay utilized premixed beads coated with anti-TNF-α, IL-6, IL-8, CCL2, CCL3, and CCL4 antibodies, which were incubated with culture supernatants in 96-well plates overnight at 4 °C with shaking. Following incubation, the beads were washed with 200 μL of supplied wash buffer followed by addition of detection antibodies (supplied with kit) and 1 h incubation at room temperature. After this, Streptavidin-phycoerythrin was added to each well and incubated at room temperature again for 30 min. Following three times washing with 200 μL wash buffer, the magnetic beads were resuspended in sheath fluid, and plates were assayed on a Luminex® 200™ system with xPONENT® software. The experimental data are presented as pg/mL. Two-way ANOVA test followed by the Bonferroni test was employed to compare the expression of cytokines and chemokines between the two cell types and a *p*-value of ≤0.05 was considered statistically significant. Data were presented as the mean relative mRNA transcript expression ± SEM.

## Data Availability

The datasets used and/or analysed during the current study available from the corresponding author on reasonable request. Gene sequences were obtained from www.ensembl.org.

## Abbreviations

CRISPR: Clustered Random Regularly Interspaced Short Palindromic Repeats
CCL2: Chemokine ligand 2
CCL4: Chemokine Ligand 4
EBV: Estimated breeding value
IFN-γ: Interferon-gamma
IL10: Interleukin 10
IL10RA: Interleukin 10 receptor alpha
IL-1α: Interleukin 1 alpha
IL-1β: Interleukin 1 beta
IL-6: Interleukin 6
JD: Johne’s disease
LPS: Lipopolysaccharide
MAC-T: Bovine mammary epithelial cell line
MAMPs: Microbe-associated Molecular Membrane Patterns
MAP: *Mycobacterium avium subsp. paratuberculosis*
RT-PCR: Real time Polymerase Chain Reaction
SOCS3: Suppressor of cytokine signaling 3
SNP: Single Nucleotide Polymorphism
sgRNA: Single guide RNA
TNF-α: Tumor necrosis factor alpha
IBD: Inflammatory bowel disease
CD: Crohn’s disease
PBMCs: Peripheral blood mononuclear cells
